# Systemic Immun e–Inflammation Index as a Predictor for Head and Neck Cancer Prognosis: A Meta-Analysis

**DOI:** 10.3389/fonc.2022.899518

**Published:** 2022-06-24

**Authors:** Yun-Ting Wang, Liang-Tseng Kuo, Hsu-Huei Weng, Cheng-Ming Hsu, Ming-Shao Tsai, Geng-He Chang, Yi-Chan Lee, Ethan I. Huang, Yao-Te Tsai

**Affiliations:** ^1^Department of Otorhinolaryngology-Head and Neck Surgery, Chang Gung Memorial Hospital, Chiayi, Taiwan; ^2^Division of Sports Medicine, Department of Orthopedic Surgery, Chang Gung Memorial Hospital, Chiayi, Taiwan; ^3^Department of Radiology, Chang Gung Memorial Hospital, Chiayi, Taiwan; ^4^Department of Otorhinolaryngology-Head and Neck Surgery, Chang Gung Memorial Hospital, Keelung, Taiwan

**Keywords:** head and neck cancer, systemic immune–inflammation index, meta-analysis, biomarker, prognosis

## Abstract

**Background:**

Studies have reported inconsistent results regarding the prognostic value of the systemic immune–inflammation index (SII) in head and neck cancer (HNC). Thus, the present meta-analysis assessed the literature on the prognostic value of SII in those with HNC.

**Methods:**

The Cochrane Library, EMBASE, and PubMed databases were searched, and study methodological quality was assessed using the Newcastle–Ottawa quality assessment scale. To determine the association of the SII with survival outcomes, pooled hazard ratios (HRs) as well as the associated 95% confidence intervals (CIs) were used. To assess the associations of the SII with clinicopathological features, the odds ratios (ORs) and corresponding 95% CIs were considered. Begg’s funnel plot and Egger’s linear regression test were used to assess publication bias.

**Results:**

A total of 12 studies that together enrolled 4369 patients with HNC were analyzed. In the pooled results, a high pretreatment SII was correlated with poorer overall survival (HR = 2.09, 95% CI = 1.62–2.70, *p* < 0.001), disease-free survival (HR = 2.79, 95% CI = 1.99−3.89, *p* < 0.001), and progression-free survival (HR = 1.80, 95% CI = 1.30−2.48, *p* < 0.001). A stratified analysis indicated that SII for overall survival was applicable regardless of tumor site, treatment modality, overall stage, sample size, SII cutoff, and method for determining the SII cutoff. Furthermore, a high SII was correlated with a more advanced T classification (OR = 1.14, 95% CI = 1.09–1.18, *p* < 0.001) and nodal metastasis (OR = 1.55, 95% CI = 1.18–2.05, *p* = 0.002) in patients with HNC.

**Conclusions:**

An elevated pretreatment SII predicts more advanced tumor and nodal status and poorer survival outcomes in cases of HNC. Because the measurement of SII is convenient and its use is cost-effective, we suggest that it can be applied by clinicians in the management of HNC.

## Introduction

In 2018, head and neck cancer (HNC) accounted for 4.6% of all cases of cancer worldwide and resulted in 430,000 deaths; the disease also entails a large economic burden (1, 2). The oral cavity is the most common tumor site, followed by the larynx, nasopharynx, oropharynx, and hypopharynx (1, 3). Cigarette smoking and alcohol consumption are leading risk factors for HNC, and 95% of histopathological diagnoses are of squamous cell carcinoma (4). At present, curative resection and definitive radiotherapy (RT) or chemoradiotherapy (CRT) are the mainstays of treatment for patients with HNC, and multidisciplinary treatment is usually required for patients with advanced disease (2). Although diagnostic and treatment modalities have improved, patients with HNC still have a poor long-term prognosis, and about 40%–60% of patients have locoregional recurrence and distant metastasis (5). Thus, cost effective and readily available prognostic biomarkers of HNC must be identified.

As indicated by an increasing number of studies, cancer-associated inflammation in a tumor microenvironment is associated with tumorigenesis, cancer progression, and an increased risk of distant metastasis (6–8). In addition, the response cells involved in systemic inflammation, such as lymphocytes, neutrophils, and platelets, serve crucial functions within the tumor microenvironment (8, 9). Thus, several inflammatory biomarkers derived from peripheral blood cells, such as the platelet-to-lymphocyte ratio (PLR) and neutrophil-to-lymphocyte ratio (NLR), have been revealed to have significant prognostic value in those with HNC (10, 11). The systemic immune–inflammation index (SII), calculated as *platelet count × neutrophil count / lymphocyte count*, is a newly proposed biomarker that has been used for the prognosis of various malignancies, such as colorectal (12), liver (13), bladder (14), lung (15), and cervical (16) malignancies. In 2018, a meta-analysis of 22 studies that recruited 7657 patients in total reported that an increased SII predicts poorer survival outcomes in various cancers (17). However, previous meta-analyses on the use of SII as a prognostic marker for cancer have failed to include studies on HNC. In addition, previous studies investigating the prognostic performance of the SII for HNC have employed relatively small sample sizes (18–29) and yielded inconsistent findings (22). For instance, several researchers have reported that the SII can be used to discriminate between patients at high versus low risk of HNC, whereas another study noted poor prognostic performance for nasopharyngeal cancer (22). Hence, this meta-analysis was performed to assess whether pretreatment SII can be used as a prognostic indicator in HNC. The correlations of the SII with the clinicopathological features of HNC was also investigated.

## Materials and Methods

### Literature Search

We performed the meta-analysis in accordance with the Preferred Reporting Items for Systematic Reviews and Meta-Analyses guidelines (30) and searched the Cochrane Library, EMBASE, and PubMed databases for papers published up to June 15, 2021. A unique search strategy was adopted for each database, as detailed in **Supplementary File 1**. We also looked up potentially relevant studies in the reference lists of the studies identified. We included studies regardless of their language of publication, research design (retrospective or prospective), or participant ethnicity. Because we analyzed only previously published data, our meta-analysis required no institutional review board approval.

### Selection Criteria

We included studies that (1) recruited patients with pathologically confirmed HNC, (2) had SII measurements based on laboratory test results before treatment, (3) provided sufficient data for the calculation of the hazard ratios (HRs) and the relevant 95% confidence intervals (CIs) for the correlations of pretreatment SII with survival outcomes, and (4) defined low and high SIIs relative to a selected cutoff value. We excluded studies that (1) were commentaries, letters, case reports, conference abstracts, or reviews; (2) were duplicate publications; (3) had insufficient data for us to calculate survival outcomes; and (4) were based on animal experiments.

### Extraction of Data and Quality Assessment

Two authors (Y-TW and Y-TT) independently reviewed eligible articles and collected data on the following: (1) publication details (publication year, name of first author, study country, participant ethnicity, study design, sample size, sex and age distribution, and follow-up duration); (2) pathological characteristics (tumor site, cancer stage at diagnosis, and clinicopathological parameters), and (3) clinical features (treatment modalities, SII cutoff value, method for determining cutoff value, survival analysis and outcome results, HRs, and 95% CIs). In case of discrepancies between the two authors, the study’s third author (L-TK) was consulted. The main outcome measures were the HRs and the relevant 95% CIs for overall survival (OS), disease-free survival (DFS), and progression-free survival (PFS), and if data on these outcome measures were unavailable, we computed them using the method proposed by Tierney and Parmar, which involves a Kaplan–Meier survival analysis (31, 32). If an included study reported both univariate and multivariate analysis results for survival analysis, the multivariate results were considered because confounding variables are accounted for. The two aforementioned authors (Y-TW and Y-TT) used the Newcastle–Ottawa scale (NOS) (33) to independently evaluate the study quality; the NOS comprises three domains pertaining to participant selection (points range: 0−4), comparability between groups (points range: 0−2), and clinical outcomes (points range: 0−3). Scores on the NOS range from 0 to 9 points, where scores of ≥ 6 indicate high methodological quality.

### Statistical Analysis

A random-effects model meta-analysis was used to integrate all outcomes because of the expected inherent heterogeneity among the eligible studies (34). The selected endpoints were the correlations of SII with DFS, PFS, and OS; the HRs and 95% CIs from the studies’ survival analyses were pooled to compute these endpoints. The correlations of the SII with clinicopathological features were assessed using pooled odds ratios (ORs) and the relevant 95% CIs. Study heterogeneity was qualitatively and quantitatively indicated if *p* < 0.1 in a Cochran’s *Q* test and if *I*^2^ > 50%, respectively. To determine the possible source of the heterogeneity, we conducted a stratified analysis in which the studies were stratified by tumor site, treatment modality, overall cancer stage, study sample size, SII cutoff value, and method for determining the SII cutoff. We used the Begg’s funnel plot asymmetry test and Egger’s linear regression test to determine publication bias (35, 36). We used Comprehensive Meta-Analysis version 3 (Biostat, Englewood, NJ, USA) for all statistical analyses, with *p* < 0.05 (two tailed) indicating significance.

## Results

### Study Selection and Characteristics of Eligible Studies

**Figure 1:**
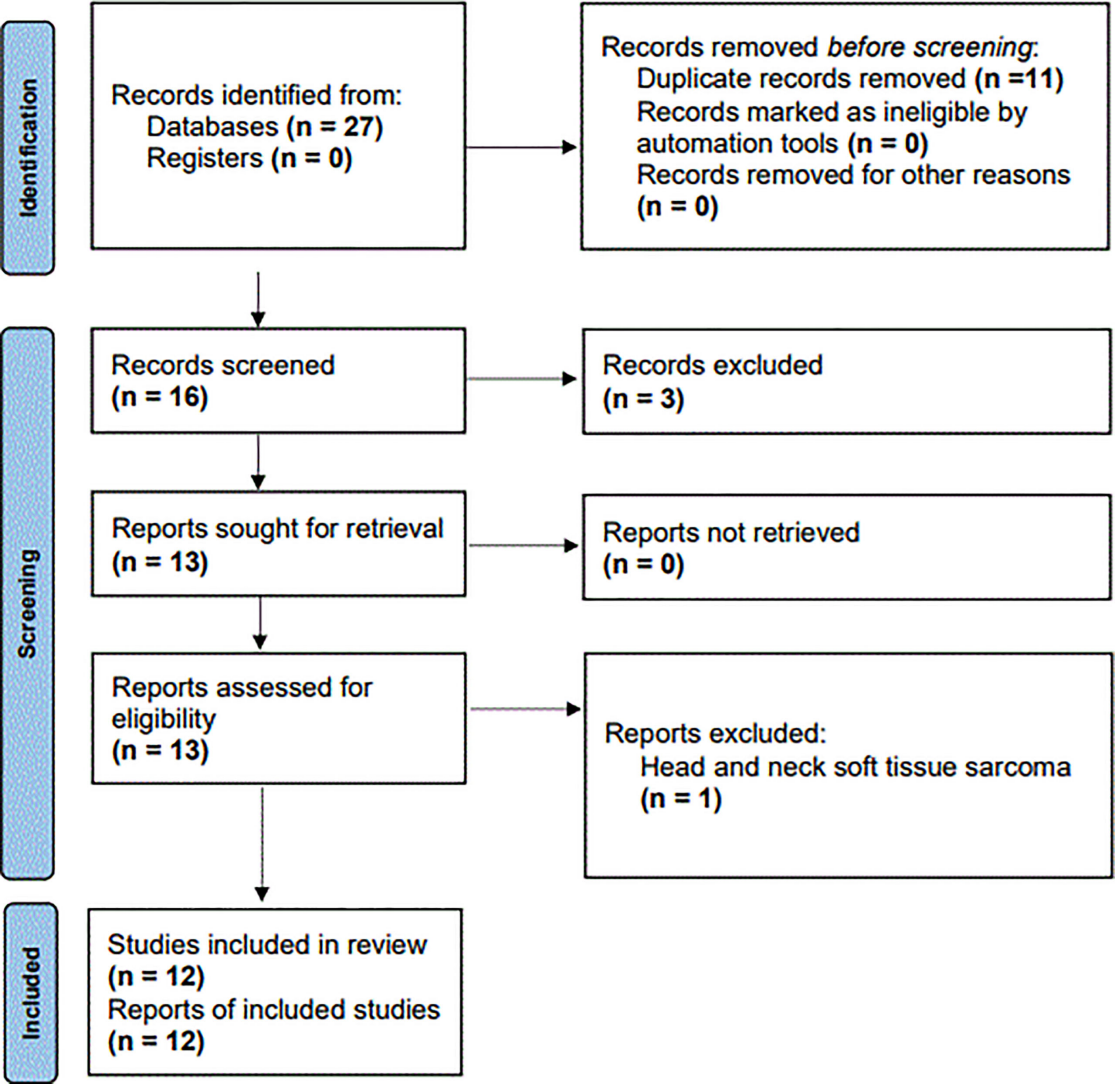
Flow diagram illustrating study selection.

**Figure 1** presents a flowchart of the study selection process. Specifically, we discovered 27 relevant studies through our initial database search. We excluded three studies after screening their abstracts and titles and 11 duplicate studies. Finally, we excluded 1 of the remaining 13 studies after reading the full texts (18–29, 37) because that study, focusing on soft tissue sarcoma of the head and neck, provided insufficient information for us to extract an HR (37). This eventually left us with 12 studies involving 4369 patients with HNC for the following meta-analysis (18–29); these studies’ baseline characteristics are presented in **Table 1**. The studies each enrolled 118 to 993 patients and were published between 2017 and 2021. Among these 12 studies, 6 (2344 patients) enrolled patients with nasopharyngeal cancer (NPC) (18, 19, 21–23, 27), 3 (1422 patients) enrolled patients with oral cavity cancer (20, 26, 28), and the remaining 3 (603 patients) enrolled patients with laryngeal cancer (24, 25, 29). The studies had samples of 118 to 993 patients (median: 318 patients); therefore, a sample size of 320 was selected as the cutoff value for sample size in the stratified analysis. Three studies focused on patients with stages III and IV HNC (21, 23, 27), and nine studies focused on patients with both early and advanced HNC (stages I−IV) (18, 21, 22, 24–29). The SII cutoffs were between 403 and 934 with a median value of 522; these cutoffs were determined using X-Tile software in two studies (20, 26), the median in one study (24), recursive-partitioning analysis in one study (21), and receiver operating characteristic curves analysis in eight studies (18, 19, 22, 23, 25, 27–29). On the basis of the median SII cutoffs in the included studies, we selected an SII cutoff of 520 for the stratified analysis.

**Table 1:** Baseline characteristics of the included studies.

First author	Published year	Country	No. of patients	Tumor site	Overallstage	Treatment	Cutoff selection	Cutoffvalue	Survival outcome	NOSScore
Jiang	2017	China	327	Nasopharynx	I−IV	RT or CRT	ROC	403	OS	8
Oei	2018	China	585	Nasopharynx	I−IV	RT or CRT	ROC	527.2	OS, PFS, DMFS	8
Diao	2018	China	309	Oral cavity	I−IV	Surgery +/- RT or CRT	X-tile software	484.5	OS, DFS	8
Lin	2019	China	243	Nasopharynx	IV	RT or CRT	RPA	930	OS, PFS	7
Zeng	2020	China	559	Nasopharynx	I−IV	RT or CRT	ROC	715.7	OS	8
Feng	2020	China	417	Nasopharynx	III−IVa	RT or CRT	ROC	488.9	OS, PFS	7
Shen	2020	China	338	Larynx	I−IV	Surgery +/- RT or CRT	Median	501.1	OS	7
Li	2020	China	147	Larynx	I−IV	Surgery +/- RT or CRT	ROC	517.6	OS, PFS	8
Lu	2020	China	120	Tongue	I−IV	Surgery +/- RT or CRT	X−tile software	569	OS, DFS	8
Xiong	2021	China	213	Nasopharynx	III−IVa	RT or CRT	ROC	402.1	OS, PFS	8
Hung	2021	Taiwan	993	Oral cavity	I−IV	Surgery +/- RT or CRT	ROC	810.6	OS, LC, RC, DC	8
Akkas	2021	Turkey	118	Larynx	I−IV	Surgery +/- RT or CRT	ROC	934	OS, DFS	8

CRT, chemoradiotherapy; DC, distant control; DFS, disease- free survival; LC, local control; MV, multivariate; NOS, Newcastle- Ottawa scale; OS, overall survival; PFS, progression- free survival; RC, regional control; ROC, receiving operating characteristics; RPA, recursive- partitioning analysis; RT, radiotherapy.

All eligible studies reported data regarding the association between the SII and OS (18–29); in particular, eight studies reported findings on the performance of the SII as a prognostic indicator for DFS and PFS (19–21, 23, 25–27, 29), and two studies reported that of the SII for distant metastasis-free survival, locoregional control, and distant control (19, 28). Findings on the prognostic value for OS or DFS and PFS were directly retrieved from all studies, and multivariate analysis was used to analyze the HRs and 95% CIs. All studies had high methodological quality (NOS score ≥ 6; median NOS score = 8; see **Supplementary File 2** for more details).

### SII and OS in HNC

**Figure 2:**
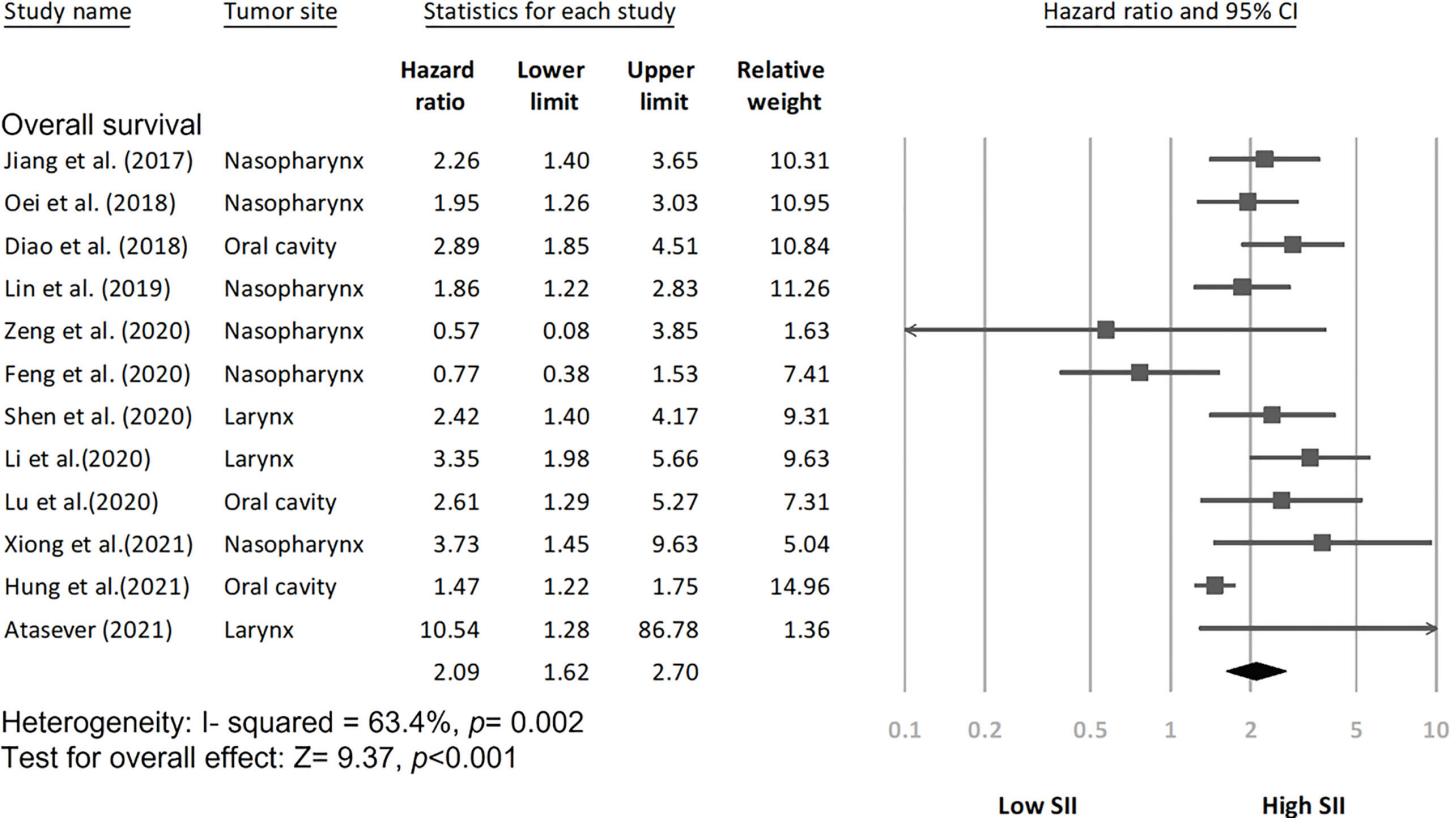
Forest plot indicating association of overall survival with SII.

According to the random-effects model, a high SII had a significant association with poorer OS relative to a low SII (HR = 2.09, 95% CI = 1.62−2.70, *p* < 0.001, **Figure 2**). Moderate heterogeneity (*I*^2^ = 63.4%, *p* = 0.002, **Figure 2**) was noted among the 12 studies (4369 patients) on the performance of the pretreatment SII as a prognostic indicator for OS. In addition, the combined results related to different tumor sites revealed that in comparison with a low SII, a high SII was correlated with poor OS in nasopharyngeal cancer (HR = 1.76, 95% CI = 1.20−2.58, *p* = 0.004), laryngeal cancer (HR = 3.12, 95% CI = 1.75−5.56, *p* < 0.001), and oral cavity cancer (HR = 2.08, 95% CI = 1.32−3.29, *p* = 0.002, **Supplementary File 3**). Stratified analysis was conducted to determine the source of heterogeneity (**Table 2**). The prognostic effect of the SII on OS remained consistent and significant across subgroups with different tumor sites (nasopharynx or oral cavity/larynx), treatment modality (RT/CRT or surgery), overall stage (mixed or advanced stages), sample size (<320 or ≥320), SII cutoff (<520 or ≥520), and method of SII cutoff determination (receiver operating characteristic curve or others). The varied heterogeneity across subgroups may have led to the moderate heterogeneity among studies reporting the OS (*I*^2^ = 63.4%; *p* = 0.002).

**Table 2:** Stratified analysis of the correlation between overall survival and SII in patients with HNC. .

Variable	Number of studies	Number of patients	Pooled HR (95%CI)	*p-*value	Heterogeneity	
					*I*^2^ (%)	P_h_
Total	12	4369	2.09 (1.62-2.7)	<0.001	63.4	0.002
Tumor sites						
Nasopharynx	6	2344	1.83 (1.45-2.3)	<0.001	52.3	0.062
Oral cavity and Larynx	6	2025	1.82 (1.57-2.11)	<0.001	74.4	0.002
Treatment						
RT or CRT	6	2344	1.83 (1.45-2.31)	0.003	52.3	0.062
Surgery +/- adjuvant therapy	6	2025	1.82 (1.57-2.11)	<0.001	74.4	0.002
Overall stage						
Mixed stages	9	3496	1.85 (1.62-2.12)	<0.001	63.3	0.005
Advanced stages	3	873	1.65 (1.18-2.31)	0.004	74.6	0.02
Sample size						
< 320	6	1150	2.64 (2.08-3.35)	<0.001	13.4	0.329
≥ 320	6	3219	1.58 (1.36-1.83)	<0.001	55.6	0.046
Cutoff of SII						
< 520	6	1751	2.4 (1.91-3.01)	<0.001	63.0	0.019
≥ 520	6	2618	1.61 (1.39-1.87)	<0.001	39.3	0.144
Methods for determining SII cutoff						
ROC	8	3359	1.67 (1.45-1.93)	<0.001	68.9	0.002
Others (X-tile, Median, RPA)	4	1010	2.35 (1.83-3.02)	<0.001	0	0.553

CRT, chemoradiotherapy; CI, confidence interval; HR, hazard ratio; ROC, receiver operating characteristic; RPA, recursive- partitioning analysis; RT, radiotherapy; SII, systemic immune-inflammation index.

### SII for DFS and PFS in HNC

**Figure 3:**
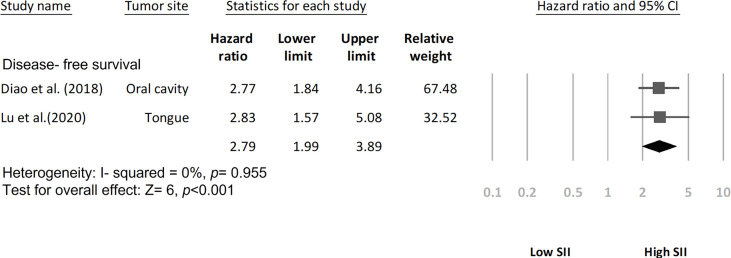
Forest plot indicating association of disease-free survival with SII.

**Figure 4:**
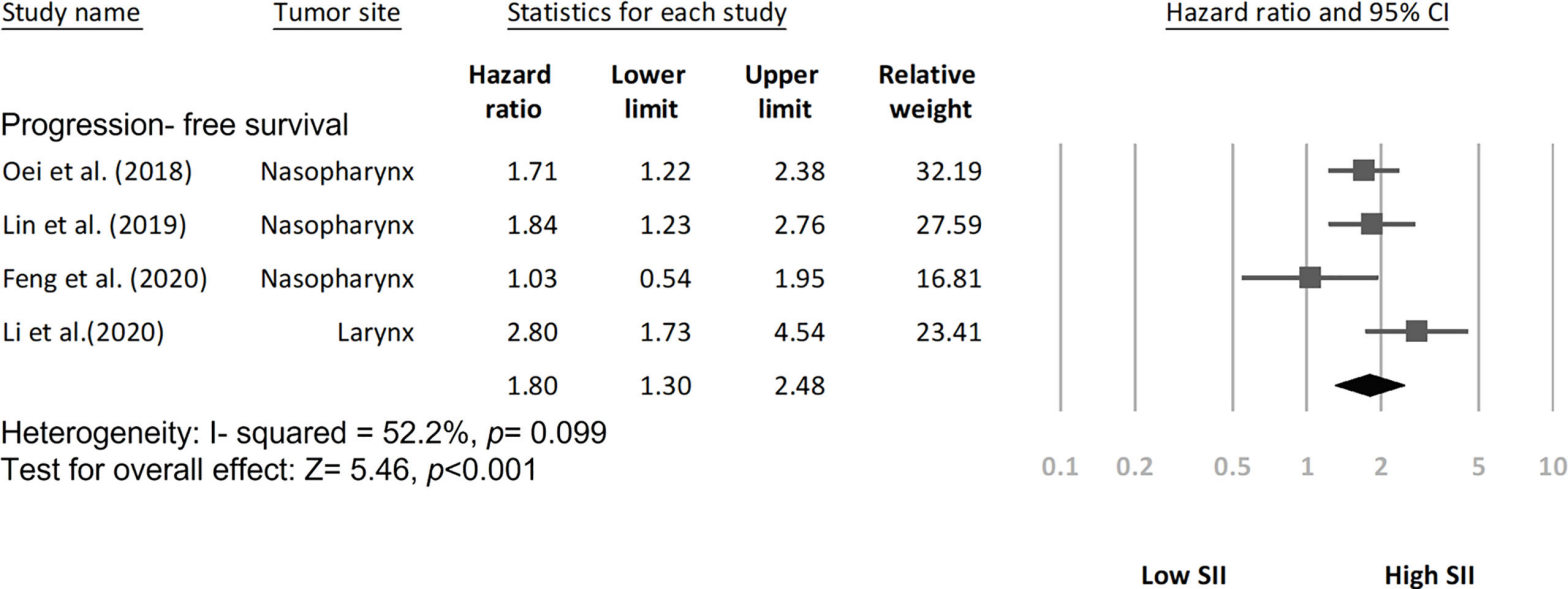
Forest plot indicating association of progression-free survival with SII.

Among the included studies, two investigated the SII–DFS relationship in HNC and enrolled 1,591 patients in total (20, 26). According to the pooled HR analysis, a high SII exhibited a significant association with poorer DFS (HR = 2.79, 95% CI = 1.99–3.89; *p* < 0.001; **Figure 3**), and low heterogeneity was noted (*I*^2^ = 0.0%; *p* = 0.955). In addition, four studies exploring the performance of pretreatment SII as a prognostic indicator of PFS were included in the analysis (19, 21, 23, 25), and the combined data indicated that a high SII had a significant association with poorer PFS (HR = 1.80, 95% CI = 1.30–2.48, *p* < 0.001; **Figure 4**) relative to a low SII. Studies on the SII–PFS association had moderate heterogeneity (*I*^2^ = 52.2%; *p* = 0.099).

### Association Between SII and Clinicopathological Features

We investigated the relationship between clinicopathological features and SII in patients with HNC and calculated the ORs and 95% CIs of SII and four clinicopathological factors, specifically T and N classifications, sex, and cancer cell differentiation. According to the synthetic results, a high pretreatment SII was significantly correlated with a more advanced T classification (T3 and T4 vs. T1 and T2; OR = 1.14, 95% CI = 1.09−1.18, *p* < 0.001) and cervical nodal metastasis (nodal metastasis vs. no nodal metastasis; OR = 1.55, 95% CI = 1.18−2.05, *p* = 0.002). However, the pooled results revealed no statistically significant correlation of SII with sex (male vs. female; OR = 0.98, 95% CI = 0.81−1.24, *p* = 0.861) or cancer cell differentiation (well differentiated vs. moderately to poorly differentiated; OR = 1.34, 95% CI = 0.75−2.41, *p* = 0.335).

### Publication Bias

**Figure 5:**
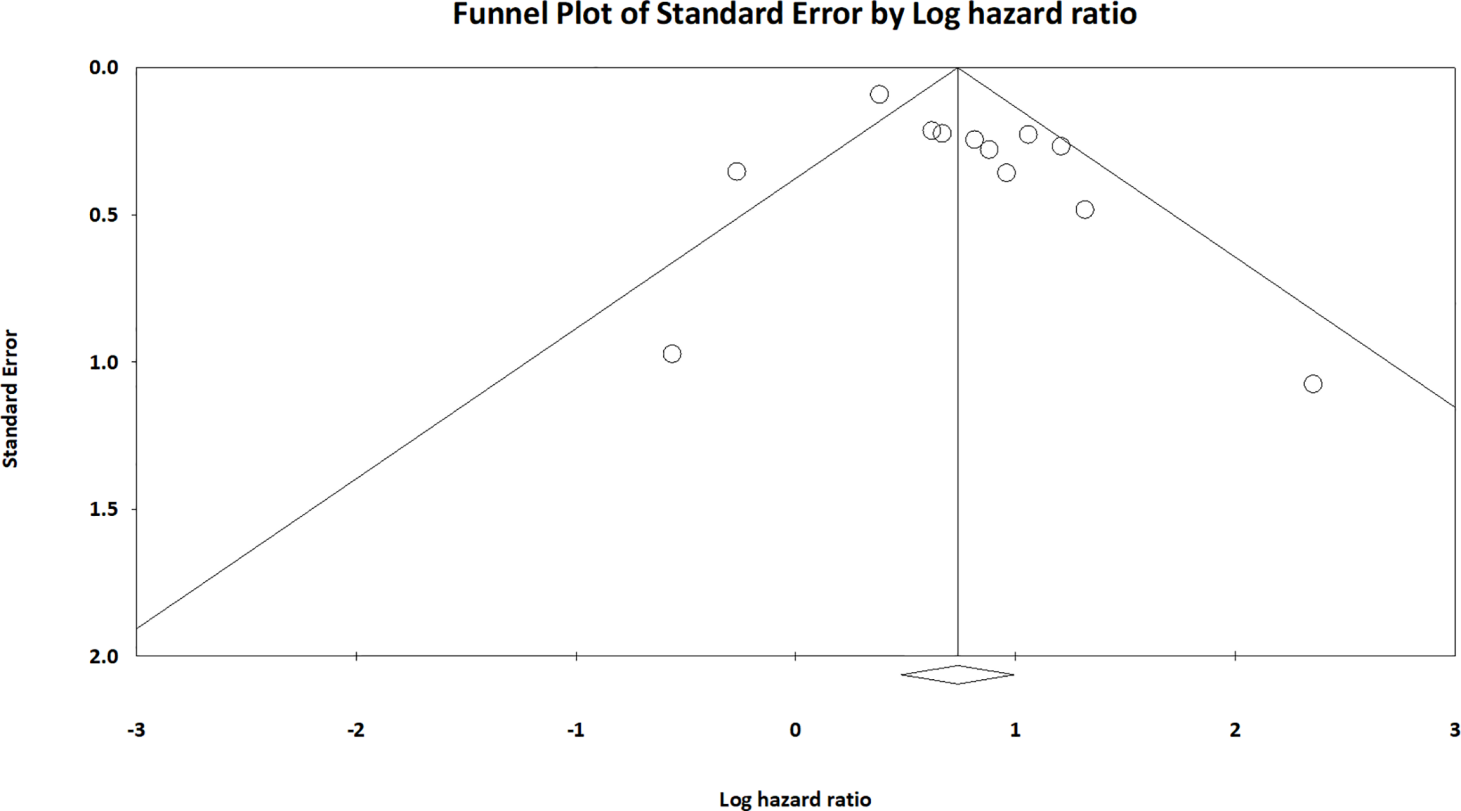
Funnel plot of publication bias test.

The funnel plot regarding publication bias for OS was symmetric, and publication bias was not significant in the Egger’s linear regression test (*p* = 0.135) and Begg’s funnel plot test (*p* = 0.217; **Figure 5**).

## Discussion

Studies have reported inconsistent results on the performance of the SII as a prognostic indicator for HNC. Meta-analyses in the literature have examined the SII’s prognostic performance in various solid tumors (17, 38), but none have included studies on HNC. Therefore, we performed a meta-analysis to assess the SII’s performance as a prognostic biomarker of various clinicopathological features of HNC. We analyzed 12 studies enrolling 4369 patients in total, and the pooled results indicate that a high pretreatment SII had significant correlations with worse PFS, DFS, and OS (HR= 1.80, 2.79 and 2.09 respectively) in patients with HNC. The stability and robustness of our results were further demonstrated in the stratified analysis, in which the SII was associated with OS regardless of tumor site, treatment, sample size, SII cutoff values, and method for determining the cutoff. The pooled results also demonstrated that a high pretreatment SII was associated with adverse pathological features of HNC, especially with respect to advanced T classification and nodal metastasis. Our results jointly indicate SII’s clinical applicability as a prognostic biomarker in patients with HNC. Because of the significant associations between pretreatment SII and aggressive clinicopathological features of HNC, our study results also suggest that the monitoring of pretreatment SII may aid in the early detection of advanced disease features and tumor progression. Extending from these findings, patients who have the elevated pretreatment SII might benefit from more aggressive treatment strategies and close follow-up, which warrants further investigation. In order to facilitate the clinical implication of SII, some researchers proposed the use of nomogram model to incorporate the cancer stages, clinicopathological factors and SII and provide accurate survival prediction in patients with HNC (26), suggesting the consideration of SII as a rational adjunct to the prognostication of patients with HNC. Because the SII can be easily and inexpensively measured using blood samples, the SII can be adopted in everyday clinical practice for personalized treatment planning with regard to HNC.

Systemic inflammatory responses play key roles in different cancer development stages, including the tumorigenesis, cancer progression, angiogenesis, and distant metastasis (39). Furthermore, in the tumor microenvironment, the relationships between inflammatory cells and cancer development are complex. Nowadays, the biomarkers based on inflammatory cells are increasingly used in clinical practice because of their high availability and cost-effectiveness. Among hematological parameters, platelet, neutrophil, and lymphocyte counts and combinations thereof, such as the PLR and NLR, have much promise as prognostic biomarkers for HNC (10, 40). Nevertheless, when only one or two parameters are accounted for, these biomarkers may be susceptible to confounding factors such as infection or liver disease. As a more comprehensive hematological biomarker, the SII combines absolute lymphocyte, neutrophil, and platelet counts and can objectively reflect changes in the numbers of these cells and, by consequence, the balance between antitumor immunity and cancer-related systemic inflammation (41). In addition, the SII has been reported to possess superior prognostic discrimination ability compared with the NLR (42) and to perform well as a prognostic indicator of a variety of malignancies, such as urological cancer, breast cancer, lung cancer, and gastric cancer (43–46). Our study results support SII’s potential as prognostic biomarker for patients with HNC, suggesting the informative role of inflammatory cells in the management of HNC.

Although scholars remain uncertain as to the molecular mechanisms explaining the correlation between the SII and HNC prognosis, several candidate mechanisms can be considered. The first potential mechanism involves the neutrophils, which are pivotal components of tumorigenesis and cancer progression because they produce various cytokines, such as interleukin 6 and 8, hepatocyte growth factor, and vascular endothelial growth factor, that induce cancer cell growth (47, 48). In addition, neutrophils can suppress the cytotoxicity of activated T lymphocytes and natural killer cells through the generation of nitric oxide and reactive oxygen species (49). Therefore, high peripheral neutrophil counts are potentially related to poor cancer survival. Another potential mechanism involves lymphocytes, which facilitate the activation of the host immune response to cancer by inhibiting the growth and proliferation of cancer cells through direct cytotoxic cell death in immune surveillance (50). Notably, the tumor-infiltrating lymphocytes serve a key function in antitumor activity; as an example, one study regarded lymphopenia to be the presentation of cytotoxic T-cell damage and inadequate antitumor immunity (51). Finally, patients with cancer often experience a state of hypercoagulation, and platelet activation may be a chemoattractant that induces tumor cell migration and enhances metastasis through the generation of bioactive proteins that are related to angiogenesis and osteoclast resorption (52). An elevated platelet count is also correlated with poor prognosis among patients with HNC (10). Therefore, a high SII suggests strong protumor activity but relatively weak anticancer immunological reaction that ultimately results in poor prognosis.

Our results also reveal that a high SII exhibits an association with aggressive clinicopathological features of HNC, including advanced T classification and nodal metastasis, which may explain why the SII can be used as a prognostic biomarker of HNC. Two mechanisms may underlie these associations. First, greater tumor burden, including nodal metastasis and tumor enlargement, may occur together with higher cancer-mediated systemic inflammation and increased neutrophil counts through cytokine production, thus leading to a higher SII value (53). Second, larger tumors may produce higher levels of proapoptotic molecules, which activate the extrinsic pathway of apoptosis and destruction of lymphocytes (54), resulting in a high SII. Further investigation into these mechanisms is required.

Meta-analyses have considered the value of the SII as a prognostic biomarker in human malignancies (44, 46). A comprehensive meta-analysis of 22 studies involving 7657 patients in total indicated that a high SII (i.e., above the cutoff) predicts worse OS, PFS, DFS, cancer-specific survival, and relapse-free survival in various cancers (17). A meta-analysis focusing on non-small-cell lung cancer reported that a high pretreatment SII was significantly correlated with poorer OS, DFS, and PFS (55), which accords with our results. Another meta-analysis of 8 studies involving 2642 patients with breast cancer demonstrated that a high SII predicts advanced T classification and nodal metastasis (44); this result is also in line with our results. The results of previous meta-analyses support our findings and further suggest the feasibility of employing the SII for HNC prognosis.

The limitations of our study should be acknowledged. First, all eligible studies were observational and retrospective studies; thus, the results might be subject to bias that affect the reliability of the study’s conclusions. Second, most studies were conducted in Asia, and the SII’s prognostic performance in non-Asian patients with HNC warrants further investigation, which will add strength to the generalizability of the study results. Third, the included studies used different methods of determining SII cutoff, which may partially explain the observed heterogeneity. Of note, despite numerous studies have demonstrated the prognostic roles of inflammatory biomarkers in various neoplasms, none of these has yet been adopted in the current treatment guidelines of HNC. Several possible scenarios may explain this observation. First, a lot of inflammation-related biomarkers had been proposed in studies of inflammation and cancer survival (56), but the lack of consensus on the optimal inflammatory biomarker and cutoff value for predicting HNC prognosis impair their clinical applicability. Second, the levels of these inflammatory biomarkers may be influenced by a variety of host conditions (e.g. infection and chronic liver disease), which may decrease the reliability of these markers and limit their use in clinical practice (57). Based on the aforementioned observations, further prospective studies with larger samples of patients from different ethnicities are required to validate our results before their use in clinical practice can be advocated.

## Conclusion

Our meta-analysis indicates that a high pretreatment SII is significantly related to worse OS, DFS, and PFS and to higher T and N classifications in patients with HNC. In addition to having favorable prognostic performance, the SII is easy and inexpensive to measure and can thus be used in clinically applications. Nonetheless, given the aforementioned limitations, our study results should be validated by further well-designed, large-scale prospective studies that include participants from a diverse range of ethnicities.

## Data Availability Statement

The original contributions presented in the study are included in the article/**Supplementary Material**. Further inquiries can be directed to the corresponding author.

## Author Contributions

Y-TW and Y-TT conceived of the study, designed the study, data interpretation and drafted the manuscript. L-TK, H-HW, and G-HC substantively revised the manuscript. C-MH and M-ST participated in the design of the study and data interpretation. Y-CL and EH participated in the statistical analysis and data interpretation. All co-authors have reviewed and approved this version of the manuscript.

## Funding

This work was supported by a grant (CMRPG6L0231) from Chiayi Chang Gung Memorial Hospital.

## Conflict of Interest

The authors declare that the research was conducted in the absence of any commercial or financial relationships that could be construed as a potential conflict of interest.

## Publisher’s Note

All claims expressed in this article are solely those of the authors and do not necessarily represent those of their affiliated organizations, or those of the publisher, the editors and the reviewers. Any product that may be evaluated in this article, or claim that may be made by its manufacturer, is not guaranteed or endorsed by the publisher.

## Supplementary Material

Supplementary File 3 Forest plots indicating associations of overall survival with SII related to different primary tumor sites.
