# Supplementary material for: Systemic Immun e–Inflammation Index as a Predictor for Head and Neck Cancer Prognosis: A Meta-Analysis
Source: Front Oncol. 2022 Jun 24;12:899518. doi: 10.3389/fonc.2022.899518 (PMC9263088; doi:10.3389/fonc.2022.899518)
Supplement: Supplementary file 1 [file Table_1.docx]

**Supplementary file 1** | Detailed search strategies.

| **CENTRAL (via Cochrane Register of Studies)** | **PubMed** | **EMBASE (Ovid)** |
| --- | --- | --- |
| #1 MeSH descriptor: [Head and Neck Neoplasms], this term only  #2 MeSH descriptor: [ Otorhinolaryngologic Neoplasms] explode all trees  #3 MeSH descriptor: [ Neck Dissection] explode all trees  #4 MeSH descriptor: [ Laryngectomy] explode all trees  #5 MeSH descriptor: [ Neoplasms] explode all trees  #6 (cancer* OR carcinoma* OR neoplas* OR tumor* OR tumour* OR malignan* OR SCCA)  #7 (“head and neck”:ti OR “head neck”:ti OR “head-neck”:ti OR “headand-neck”:ti OR oral:ti OR oropharyn*:ti OR hypopharyn*:ti or laryn*:ti OR nasopharyn*:ti OR pharyn*:ti OR throat:ti OR mouth:ti)  #8 (#5 OR #6) AND #7  #9 HNSCC OR SCCHN OR laryngectom* OR (neck AND dissect*)  #10 #1 OR #2 OR #3 OR #4 OR #8 OR #9  #11 “Systemic immune-inflammation index” or SII  #12 #10 and #11 | #1 ("Head and Neck Neoplasms/surgery"[Mesh])  #2 "Otorhinolaryngologic Neoplasms/surgery"[Mesh]  #3 ("Head and Neck Neoplasms"[Mesh])  #4 "Otorhinolaryngologic Neoplasms"[Mesh]  #5 ("head neck" OR head‐neck OR "head and neck" OR head‐and‐neck[Title/Abstract])  #6"larynx"[Title/Abstract] OR "laryngeal"[Title/Abstract] OR "glottis"[Title/Abstract] OR "glottic"[Title/Abstract] OR "oral cavity"[Title/Abstract] OR "nasopharynx"[Title/Abstract] OR "nasopharyngeal"[Title/Abstract] OR "hypopharynx"[Title/Abstract] OR "hypopharyngeal"[Title/Abstract] OR "pharynx"[Title/Abstract] OR "pharyngeal"[Title/Abstract] OR "parapharyngeal"[Title/Abstract] OR "mouth"[Title/Abstract]  #7(#5 OR #6)  #8"cancer*"[Title/Abstract] OR "carcinoma*"[Title/Abstract] OR "neoplasm*"[Title/Abstract] OR "tumor*"[Title/Abstract] OR "tumour*"[Title/Abstract] OR "metastas*"[Title/Abstract]  #9 "Neoplasms"[Mesh]  #10(#8 OR#9)  #11(#7 AND #10)  #12"systemic immune inflammation index"[Title/Abstract] OR "sii"[Title/Abstract]  #13(#11 AND #12) | #1 "Head and Neck Neoplasms"/  #2 Otorhinolaryngologic Neoplasms/  #3 *Neck Dissection/  #4 “Laryngectomy”/  #5 (HNSCC OR SCCHN OR laryngectom* OR (neck AND dissect*) ).mp.  #6 Neoplasms/OR (cancer OR cancers OR cancerous OR carcinoma* OR neoplas* OR tumor* OR tumour* OR malignan* OR SCCA).mp.  #7 (“head and neck” OR “head neck” OR “head-neck” OR “head-and-neck” OR oral OR oropharyn* OR hypopharyn* or laryn* OR nasopharyn* OR pharyn* OR throat OR mouth) .m_titl.  #8 #6 AND #7  #9 #1 OR #2 OR #3 OR #4 OR #5 OR #8  #10 “Systemic immune inflammation index”OR SII  #11#9 AND #10 |
