# Supplementary material for: Systemic Immun e–Inflammation Index as a Predictor for Head and Neck Cancer Prognosis: A Meta-Analysis
Source: Front Oncol. 2022 Jun 24;12:899518. doi: 10.3389/fonc.2022.899518 (PMC9263088; doi:10.3389/fonc.2022.899518)
Supplement: Supplementary file 2 [file Table_2.docx]

**Supplementary file 2** | Indices of methodological quality of the included studies according to the Newcastle–Ottawa Quality Assessment Scale.

| Study | Selection | Comparability | Outcome |
| --- | --- | --- | --- |
| Jiang et al. (2017) | **🟑🟑🟑** | **🟑🟑** | **🟑🟑🟑** |
| Oei et al. (2018) | **🟑🟑🟑** | **🟑🟑** | **🟑🟑🟑** |
| Diao et al. (2018) | **🟑🟑🟑** | **🟑🟑** | **🟑🟑🟑** |
| Lin et al. (2019) | **🟑🟑🟑** | **🟑** | **🟑🟑🟑** |
| Zeng et al. (2020) | **🟑🟑🟑** | **🟑🟑** | **🟑🟑🟑** |
| Feng et al. (2020) | **🟑🟑🟑** | **🟑🟑** | **🟑🟑** |
| Shen et al. (2020) | **🟑🟑🟑** | **🟑🟑** | **🟑🟑** |
| Li et al. (2020) | **🟑🟑🟑** | **🟑🟑** | **🟑🟑🟑** |
| Lu et al. (2020) | **🟑🟑🟑** | **🟑🟑** | **🟑🟑🟑** |
| Xiong et al. (2021) | **🟑🟑🟑** | **🟑🟑** | **🟑🟑🟑** |
| Hung et al. (2021) | **🟑🟑🟑** | **🟑🟑** | **🟑🟑🟑** |
| Atasever et al. (2021) | **🟑🟑🟑** | **🟑🟑** | **🟑🟑🟑** |
