# Supplementary figures and images for: Systemic Immun e–Inflammation Index as a Predictor for Head and Neck Cancer Prognosis: A Meta-Analysis
Source: Front Oncol. 2022 Jun 24;12:899518. doi: 10.3389/fonc.2022.899518 (PMC9263088; doi:10.3389/fonc.2022.899518)

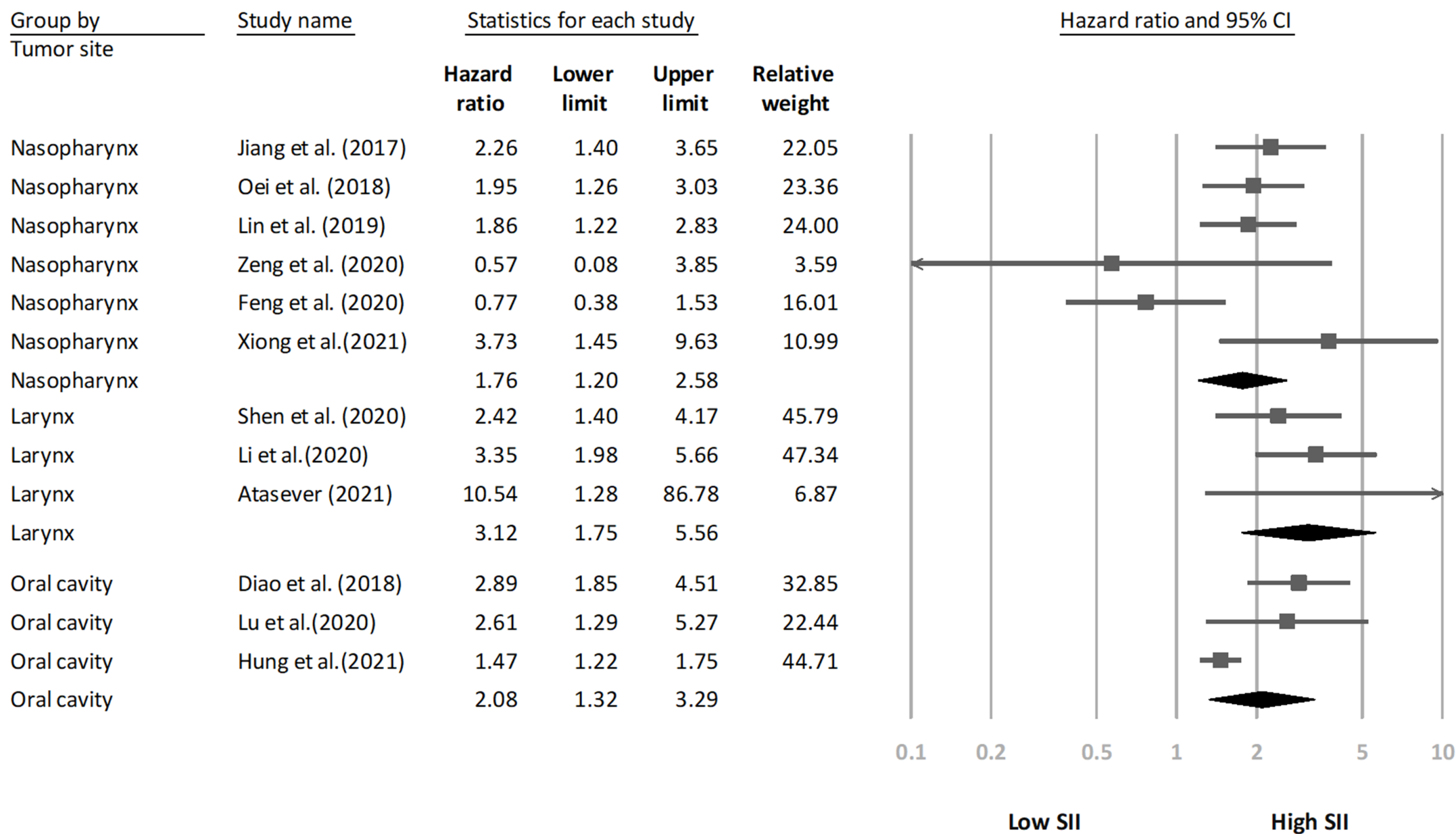

Supplement: Supplementary File 3 — Forest plots indicating associations of overall survival with SII related to different primary tumor sites. [file DataSheet_1.pdf]
